# Crystal Structure of the N-Terminal RNA Recognition Motif of mRNA Decay Regulator AUF1

**DOI:** 10.1155/2016/3286191

**Published:** 2016-06-29

**Authors:** Young Jun Choi, Je-Hyun Yoon, Jeong Ho Chang

**Affiliations:** ^1^Department of Biology Education, Kyungpook National University, Daegu 41566, Republic of Korea; ^2^Department of Biochemistry and Molecular Biology, Medical University of South Carolina, Charleston, SC 29425, USA

## Abstract

AU-rich element binding/degradation factor 1 (AUF1) plays a role in destabilizing mRNAs by forming complexes with AU-rich elements (ARE) in the 3′-untranslated regions. Multiple AUF1-ARE complexes regulate the translation of encoded products related to the cell cycle, apoptosis, and inflammation. AUF1 contains two tandem RNA recognition motifs (RRM) and a Gln- (Q-) rich domain in their C-terminal region. To observe how the two RRMs are involved in recognizing ARE, we obtained the AUF1-p37 protein covering the two RRMs. However, only N-terminal RRM (RRM1) was crystallized and its structure was determined at 1.7 Å resolution. It appears that the RRM1 and RRM2 separated before crystallization. To demonstrate which factors affect the separate RRM1-2, we performed limited proteolysis using trypsin. The results indicated that the intact proteins were cleaved by unknown proteases that were associated with them prior to crystallization. In comparison with each of the monomers, the conformations of the *β*2-*β*3 loops were highly variable. Furthermore, a comparison with the RRM1-2 structures of HuR and hnRNP A1 revealed that a dimer of RRM1 could be one of the possible conformations of RRM1-2. Our data may provide a guidance for further structural investigations of AUF1 tandem RRM repeat and its mode of ARE binding.

## 1. Introduction

Regulation of mRNA stability is one of the most important mechanisms of gene expression control, and it is related to various biological processes including the cell cycle, inflammation, oncogenesis, and apoptosis [1, 2]. Many proteins regulate mRNA stability by interacting with the AU-rich elements (AREs) in the 3′-untranslated regions (3′-UTRs) of mRNAs [3]. AREs are the most widespread* cis*-regulatory elements; they have a core AUUUA pentameric sequence within a U-rich region, and their size varies between 40 and 150 nucleotides [4]. They function by forming ribonucleoprotein (RNP) complexes with a series of ARE-binding factors. Although more than 20 ARE-binding factors have been identified, few have been functionally characterized [3, 5].

Heterogeneous nuclear ribonucleoprotein D0 (hnRNP D0), also known as AU-rich element RNA-binding protein 1 (AUF1), is a well-characterized ARE-binding factor that destabilizes mRNA, while human (Hu) antigen proteins of the ELAV (embryonic lethal, abnormal vision) family are known to stabilize mRNA. Human antigen protein R (HuR), a member of the ELAV family, contains three RNA recognition motifs (RRMs) and an inserted hinge region that includes a nuclear-cytoplasmic shuttling sequence for bidirectional transport between the nucleus and cytoplasm [6, 7]. Therefore, HuR affects positive regulation of posttranscriptional gene expression by exerting a stabilizing influence on labile mRNA [8, 9].

Several reports have shown that AUF1 and HuR functionally interact with miRNAs [10, 11]. For example, AUF1 directly binds to let-7b miRNA, which promotes its interaction with Argonaute2 [12]. Moreover, electrophoretic mobility shift assay with a mutant AUF1 showed that Cys207 is critical for binding to let-7b. These results suggest that AUF1 contributes to efficient targeting of mRNA decay through enhancing let-7b transfer to Argonaute2. Some of long (intergenic) noncoding RNAs such as UFC1 and HOTAIR also interact with HuR [13, 14]. These interactions support the fact that HuR regulates the function of long noncoding RNAs that are involved in oncogenesis and ubiquitin-mediated proteolysis.

AUF1 has four isoforms, p37, p40, p42, and p45, generated by alternative splicing [15]. The longest form, AUF1-p45, contains exon2 (19 amino acids) and exon7 (49 amino acids), while AUF1-p37 has no exon. AUF1-p40 and AUF1-p42 contain exon2 and exon7, respectively. Those isoforms have different ARE-binding affinities, with AUF1-p37 exhibiting the highest and AUF1-p40 the lowest affinity [15]. Thus, different expression patterns of the AUF1 isoforms may lead to different mRNA decay rates that could support AUF1 isoform-specific or cell type-specific regulation of gene expression [16]. For example, overexpression of AUF1-p37 selectively degrades ARE-containing mRNA in various cells, while the p40 isoform positively regulates interleukin-10 expression in monocytes [17, 18]. AUF1-p42 specifically suppresses FGF9 mRNA stability and the p45 isoform selectively binds to estrogen receptor mRNA to upregulate gene expression [19, 20].

**Figure 1:**
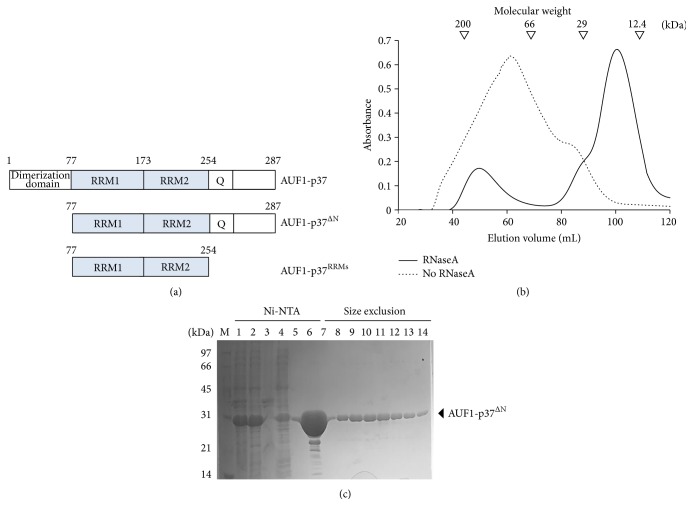
Preparation of the AUF1-p37^RRMs^ protein. (a) Domain organization of AUF1-p37 and truncated constructs. (b) Chromatogram of size-exclusion chromatography for AUF1-p37^ΔN^. The dotted line indicates a chromatogram in the absence of RNaseA. The black line represents the peak chromatogram in the presence of RNaseA. The standard molecular masses for SEC experiments were obtained from the following proteins: *β*-amylase, 200 kDa; albumin, 66 kDa; carbonic anhydrase, 29 kDa; and cytochrome C, 12.4 kDa. (c) Sodium dodecyl sulfate polyacrylamide gel electrophoresis (SDS-PAGE) of the size-exclusion chromatographic fractions, as shown in (b). The labels above the gel indicate: M, low-range marker (Bio-Rad); 1, whole-cell fraction; 2, soluble fraction; 3, insoluble fraction; 4, flow-through fraction; 5, wash fraction with 25 mM imidazole; 6, elution fraction with 250 mM imidazole; 7–14, fractions from the size-exclusion chromatography.

AUF1 contains two tandem RRMs and an adjacent Q-rich motif (Figure 1(a)). Extensive studies have been performed to determine the mechanisms by which AREs mediate mRNA decay by AUF1. However, structural information on AUF1 is currently very limited, although each of the N- and C-terminal RRM structures has been determined by NMR spectroscopy [21, 22]. Since both RRM1 and RRM2 should be involved in ARE binding, a structure containing both tandem N- and C-terminal RRMs is necessary for the investigation of the mechanism by which AUF1 binds to ARE. Accordingly, we succeeded in purifying AUF1-p37 with concomitant RNase treatment and attempted to crystallize the tandem RRMs. Although we unexpectedly observed the crystallographic dimer of RRM1, comparison with other RRM-containing structures may provide a guidance for further investigations to elucidate a real arrangement of the tandem RRMs.

## 2. Materials and Methods

### 2.1. Cloning, Expression, and Protein Purification

The amplified human AUF1-p37 gene (UniProt ID Q14103) was inserted into the pET-30a plasmid (Invitrogen, Carlsbad, CA, USA) via* Nde*I and* Xho*I restriction sites. The recombinant gene was confirmed by DNA sequencing. The resulting expression vector pET-30a:AUF1-p37 was transformed into* Escherichia coli* Rosetta (DE3) cells, which were then grown at 37°C in Luria-Bertani medium containing 50 *μ*g/mL kanamycin and 35 *μ*g/mL chloramphenicol until the optical density measured at 600 nm (OD_600_) reached ~0.7. After induction with 0.4 mM isopropyl *β*-D-1-thiogalactopyranoside at 20°C for a further 15 h, the cells were harvested by centrifugation at 5000 ×g at 277 K. All subsequent truncated AUF1-p37 constructs were generated by the same procedure.

The C-terminal 6xHis-tagged protein of full-length AUF1-p37 was purified as suggested. The harvested cells were resuspended in ice-cold buffer A (25 mM Tris–HCl (pH 8.0), 200 mM NaCl) with 200 *μ*g of RNase A (Sigma-Aldrich, St. Louis, MO, USA; R6513) to remove nonspecifically bound RNAs, and lysed by sonication. The lysate was centrifuged at 15,000 ×g for 40 min and the supernatant was loaded onto Ni–NTA resin (Qiagen, Valencia, CA, USA) equilibrated with buffer A. After washing with buffer A containing 25 mM imidazole, the bound protein was eluted in one step using 25 mM Tris–HCl (pH 8.0), 150 mM NaCl, and 250 mM imidazole. Prior to further purification, an additional 300 *μ*g of RNase A was used to remove nonspecifically bound RNA, then the sample was dialyzed overnight against 25 mM Tris–HCl (pH 8.0), 150 mM NaCl, and 30 mM *β*-mercaptoethanol at 4°C. The protein was further purified using a HiPrep 16/60 Sephacryl S-300 HR (GE Healthcare, Little Chalfont, UK) column equilibrated in 25 mM Tris–HCl (pH 7.5) and 150 mM NaCl. Fractions containing AUF1-p37 were pooled and concentrated to 25 mg/mL. The production procedure for the AUF1-p37^ΔN^ (residues 77–287) and AUF1-p37^RRMs^ (residues 77–257) proteins was identical to that for the full-length AUF1-p37. The measured concentrations of AUF1-p37^ΔN^ and AUF1-p37^RRMs^ by Bradford assays were 95 mg/mL and 120 mg/mL, respectively. Aliquots were flash-frozen in liquid nitrogen and stored at −80°C.

### 2.2. Crystallization

Crystal screening of the AUF1-p37^RRMs^ and AUF1-p37^ΔN^ proteins was performed by the sitting-drop vapor-diffusion method in 96-well sitting-drop crystallization plates (Art Robbins Instruments) with over 600 conditions by the sparse-matrix method at 20°C [23]. For screening, 1 *μ*L protein solution was mixed with 1 *μ*L reservoir solution and equilibrated against 50 *μ*L reservoir solution. Initial crystals of AUF1-p37^RRMs^ were obtained from Wizard I condition number 21 consisting of 0.1 M 4-(2-hydroxyethyl)-1-piperazineethanesulfonic acid (HEPES; pH 7.5), 20% (w/v) polyethylene glycol 8 K, and Wizard I condition number 16 consisting of 2.5 M sodium chloride and 0.1 M sodium potassium phosphate (pH 6.2). To obtain crystals suitable for X-ray diffraction, the crystallization conditions were further optimized by varying the concentration of protein, the pH, and the precipitants using the hanging-drop vapor-diffusion method. The optimized thin-plate crystals with dimensions of approximately 0.3 × 0.2 × 0.02 mm were obtained after 1 week under the conditions of 30% (w/v) polyethylene glycol 3350 and HEPES (pH 6.5) with a protein concentration of 120 mg/mL.

### 2.3. Data Collection and Structure Determination

For X-ray data collection, a single crystal was immersed briefly in a reservoir solution containing 25% glycerol as a cryoprotectant and immediately flash-cooled in a 100 K nitrogen stream. Native X-ray diffraction data were collected using an ADSC Q315r CCD detector on beamline 7A at the Pohang Accelerator Laboratory (PAL; Republic of Korea) using 1° oscillations with a crystal-to-detector distance of 200 mm. The crystal was exposed for 1 s per image. A data set was collected at 1.7 Å resolution from a single crystal. The data were indexed and scaled using the HKL-2000 software package [24]. Statistics for the diffraction data collection and processing are given in Table 1.

Phase determination by molecular replacement was attempted using the programs MOLREP [25] and Phaser [26]. We performed an initial search using the NMR structures of AUF1-RRM1 (PDB entry 1HD0 [21]) and AUF1-RRM2 (PDB entry 1X0F [27]) as starting models. However, no correct solution was obtained using either of the programs for unknown reasons. To overcome the phasing problem, we also produced selenomethionine-substituted crystals using similar conditions. Selenium site searching and experimental single-wavelength anomalous diffraction (SAD) phasing were calculated using the AutoSol pipeline in the Phenix program [28]. Model building was performed using the Coot program [29]. The model was further refined using CCP4 refmac5 [30]. The data processing and refinement statistics are summarized in Table 1.

### 2.4. Limited Proteolysis

AUF1-p37^RRMs^ (20 *μ*g) was digested with trypsin or subtilisin. Digestions were performed in a buffer containing 25 mM Tric-HCl, pH 8.0, 200 mM NaCl, and 1 mM dithiothreitol using the various amounts of either trypsin at 10°C for 90 min or subtilisin at 4°C for 60 min. The reactions were stopped by addition of final 1 mM phenylmethylsulfonyl fluoride (PMSF; Sigma-Aldrich, St. Louis, MO, USA; 78830) and SDS sample buffer. Samples were boiling at 100°C for 5 min prior to be fractionated by SDS-PAGE and then stained with Coomassie Brilliant Blue R (Sigma-Aldrich, St. Louis, MO, USA; 27816).

## 3. Results and Discussion

### 3.1. Preparation of AUF1-p37 Protein and Structure Determination

Initially, we succeeded in solubilizing the full-length AUF1-p37 protein in an* E. coli* expression system; however, during the gel filtration step, the chromatogram showed an extremely broad main peak and its position indicated a highly multimeric form, which is not suitable for target protein crystallization. We therefore designed several N- or C-terminally truncated constructs based on secondary structure prediction analysis [31]. Through extensive screenings, the two recombinant proteins of AUF1-p37^ΔN^ (residues 77–287) and AUF1-p37^RRMs^ (residues 77–254) were successfully overexpressed and solubilized (Figure 1(a)). However, a broad peak of the multimeric form was still detected in the size-exclusion step, which was similar to that of the full-length protein (Figure 1(b)).

Since the two AUF1-p37 constructs contain two tandem RRMs, we considered the possibility of nonspecific bindings of endogenous* E. coli* RNAs. To verify this, we measured the 260/280 ratios after Ni-NTA affinity analysis and gel filtration and found them to be 1.762 and 1.471, respectively, indicating that fragmented intrinsic* E. coli* RNAs were bound to the target protein (data not shown). We expected that the heterogeneously bound RNA would negatively affect the crystal packing of the protein. Therefore, to remove the nonspecifically bound RNAs, we included 200 *μ*g of RNase A during the cell lysis step and an additional 300 *μ*g was supplied after affinity chromatography. The RNA fragments were then removed by dialyzing against the gel filtration buffer. As a result, the gel filtration chromatogram showed a symmetrical peak at the monomeric position (Figure 1(b)), and the 260/280 ratio of the peak material was 0.68. The homogeneity was confirmed by sodium dodecyl sulfate polyacrylamide gel electrophoresis (SDS-PAGE), in which a single band with about 97% purity was observed (Figure 1(c)).

The first crystallization attempt was performed using both the purified AUF1-p37^ΔN^ and AUF1-p37^RRMs^ subjected to over 600 conditions from sparse-matrix screenings. Optimized thin-plate crystals were obtained from the AUF1-p37^RRMs^ protein using 30% (w/v) polyethylene glycol 3350 and HEPES (pH 6.5) after 1 week. A selected AUF1-p37^RRMs^ crystal was diffracted to a resolution of 1.7 Å and belonged to space group* P*2_1_2_1_2_1_. Phase determination by molecular replacement was attempted using the programs MOLREP [25] and Phaser [26], with an initial search using the NMR structures of AUF1-RRM1 (PDB entry 1HD0) and AUF1-RRM2 (PDB entry 1X0F) as starting models. Sequence identity between AUF1-RRM1 and AUF1-RRM2 is 41.6% over 77 residues. However, no correct solution was obtained using either program for unknown reasons. One strong possibility is that the search models were not properly prepared because we never expected the crystals to contain only the AUF1-RRM1 fragment (see Section 3.2). Consequently, we produced selenomethionine-substituted crystals using similar conditions and experimental SAD phasing succeeded using the AutoSol pipeline in the Phenix program [28].

### 3.2. Overall Structure of AUF1-p37^RRM1^

**Figure 2:**
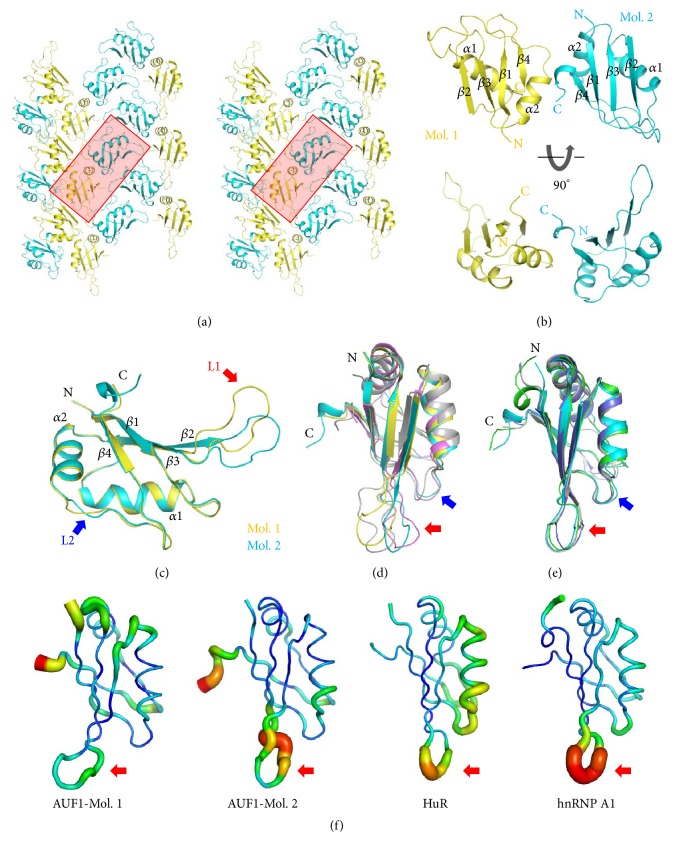
Overall structure of AUF1-p37^RRM1^. (a) Stereo diagram of* P*2_1_2_1_2_1_ symmetry packings of AUF1-p37^RRM1^. The highlighted red box represents the dimeric RRM1 molecules in the asymmetric unit. (b) Overall structure of dimeric AUF1-p37^RRM1^. The two RRM1 molecules are colored in yellow and cyan. The 90° rotated view along the *x*-axis is shown in the lower panel. (c) Overlay of the two RRM1 molecules. Conformational differences of the L1 (*β*2-*β*3) and L2 (*α*2-*β*4) loops are indicated by red and blue arrows, respectively. (d) Overlay of the two RRM1 molecules with the RRM1 (pink) and RRM2 (gray) molecules from the NMR structures. The L1 and L2 loops are indicated as shown in (c). (e) Overlay of RRM1-Mol. 2 (cyan) with hnRNP A1-RRM1 (green; PDB entry 2UP1), HuR-RRM1 apo (blue; PDB entry 4EGL), and HuR-RRM1-RNA (gray; PDB entries 4ED5). The L1 and L2 loops are indicated as shown in (c). (f)* B*-factor presentations of the RRM1 from two monomers of AUF1, HuR, and hnRNP A1, as indicated below. The* B*-factor represents the dynamic mobility of the different resolved parts within the structure. The thicker lines with warmer colors indicate higher mobility. Each of the L1 loops is represented by red arrows.

Once we had obtained an initial electron density map, two separated domains were clearly observed in the asymmetric unit (Figure 2(a)). Because we assumed that the asymmetric unit contains one molecule of AUF1-p37^RRMs^, we initially thought that monomeric RRM1 and RRM2 could be modeled. However, after refinement we realized that the two models of RRMs were identical because they had the same sequences as RRM1. The monomeric RRM1 molecule shows a typical RRM structure that contains *αβ* sandwich with a *βαββαβ* topology (Figure 2(b)). Each of the two molecules has a pseudo twofold symmetry along the *z*-axis. The PISA analysis, based on the calculated interface area of 374.2 Å^2^ for the two RRM1 molecules, indicates that the arrangement of RRM1 dimer is most likely generated by crystallographic packing [32]. However, as shown in the previous report that there is no interaction between the linked RRM1 and RRM2 of HuR, the two AUF1 RRM1 molecules are not necessary to be interacted with each other [33]. The two RRM1 molecules are easily superimposed with a root-mean-square deviation (RMSD) of 1.80 Å for 79  C_*α*_, except for the loops *β*2*β*3 (L1) and *α*2*β*4 (L2) (Figure 2(c)). In addition, the second molecule (Mol. 2) has three more residues at its C-terminus which partially contribute to the formation of a half-turn helix. In a comparison of the NMR structures of AUF1-RRM1 and -RRM2 with the two monomers, the two loops had different conformations (Figure 2(d)). Thus, the twenty refined main chains from the NMR structures of AUF1-RRM1 and -RRM2 support variation of the L1 loop conformation [21, 22]. However, the crystal structures of hnRNP A1-RRM1 (PDB entry 2UP1) and HuR-RRM1 (PDB entries 4ED5 and 4EGL) show very similar conformations to AUF1-RRM1 Mol. 2 (Figure 2(e)). These results may indicate that the variable conformations of the L1 and L2 loops are a feature of AUF1. Moreover, the L1 loop seems to be involved in substrate specificity [21, 34]. To assess flexibility of the L1 loop in other RRM1 molecules, we compared the* B*-factor of the L1 loop in AUF1, HuR, and hnRNP A1 (Figure 2(f)). Except for AUF1-Mol 1, most structures had a high* B*-factor compared with other regions, which indicates that the L1 loops are intrinsically flexible. Therefore, the L1 loop is generally flexible and could be involved in specific recognition in RRM-containing proteins.

### 3.3. Proteolytic Cleavage of AUF1-p37

**Figure 3:**
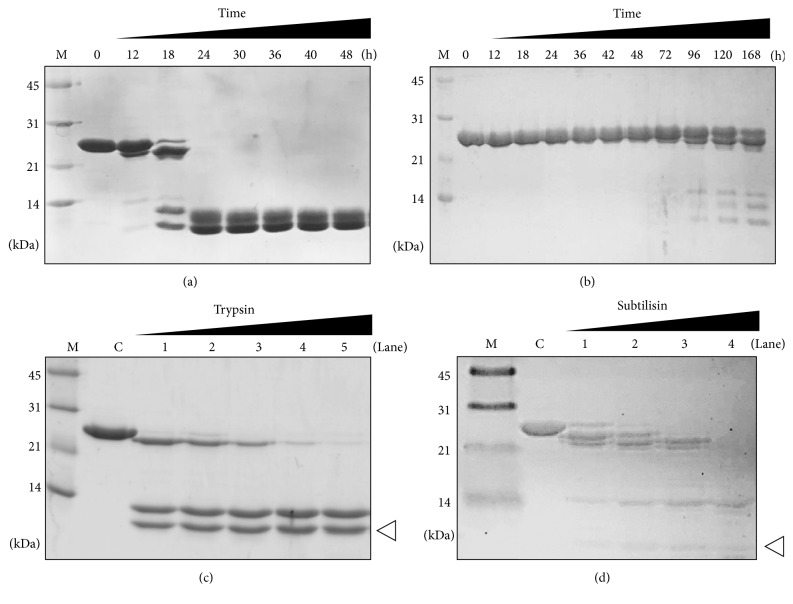
Proteolytic cleavage of AUF1-p37^RRMs^. (a) Time-course cleavage pattern of AUF1-p37^RRMs^ during 48 h at room temperature. The protein was cleaved into two small fragments (~10 kDa) after 18 h. Each of the time points is indicated above the gel. “M” and “0” indicate the low-range size marker (Bio-Rad) and the protein not incubated at room temperature (control), respectively. (b) Time-course cleavage pattern of AUF1-p37^RRMs^ in the presence of 0.2 mM PMSF during 5 days at room temperature. Each of the time points by hours is indicated above the gel. “M” and “0” indicate the low-range size marker (Bio-Rad) and the protein not incubated at room temperature (control), respectively. (c) Limited proteolysis of AUF1-p37^RRMs^ by trypsin in a concentration-dependent manner at 10°C for 90 min. “M” and “C” indicate the marker as shown in (a) and the nontrypsin-treated control, respectively. Labels 1 to 5 represent the ratios of trypsin to AUF1-p37^RRMs^ protein: label 1, 1/2000; label 2, 1/1500; label 3, 1/1000; label 4, 1/500; and label 5, 1/300. The possible RRM1 fragment is indicated by a white arrow head. (d) Limited proteolysis of AUF1-p37^RRMs^ by subtilisin in a concentration-dependent manner at 4°C for 60 min. “M” and “C” indicate the marker as shown in (a) and the nonsubtilisin-treated control, respectively. Labels 1 to 4 represent the ratios of subtilisin to AUF1-p37^RRMs^ protein: label 1, 1/2000; label 2, 1/1500; label 3, 1/1000; and label 4, 1/500. The possible RRM1 fragment is indicated by a white arrow head.

Because we did not expect that only RRM1 would be crystallized from AUF1-p37^2RRMs^, we investigated whether the AUF1-p37^2RRMs^ protein spontaneously cleaves RRM1 and RRM2 before crystallization. The purified AUF1-p37^2RRMs^ was time-course incubated at room temperature for up to 48 h (Figure 3(a)). Cleavage of the protein was started at 12 h and completed 24 h later. Two separated bands are clearly shown below 14 kDa and may represent RRM1 (lower band) and RRM2 (upper band). Since the crystals usually appeared at least 3 days later, the AUF1-p37^2RRMs^ was cleaved prior to the crystallization of RRM1. To confirm the cleavage was due to contamination of serine proteases, the protein was time-course incubated in the presence of 0.5 mM of protease inhibitor PMSF (Figure 3(b)). Moreover, to determine how the protein was cleaved, we performed limited proteolysis using serine proteases as trypsin and subtilisin for 90 min at 10°C. The protein was readily digested by 1/2000 trypsin and produced three bands: one was shifted slightly lower and two were well below 14 kDa (Figure 3(c)). The slightly shifted band may represent the fragment cleaved approximately 10 residues from the N- or C-terminus, because basic residues are found at the 13th (Lysine) and 10th (Arginine) positions from the N- and C-terminus, respectively. This band disappeared completely at higher concentrations of trypsin (1/300 ratio), but the two small bands increased in intensity. Interestingly, compared with the time-course incubation results in Figure 3(a), the two bands were very similar in size. Digestion with subtilisin produced several slightly shifted bands that were similar to those of trypsin (Figure 3(d)). In addition, two major bands were detected: one at ~14 kDa and one far below 14 kDa. Based on the previous results, the lowest band may represent the RRM1 fragment. Because subtilisin has broader specificity than trypsin, it is possible that additional cleavage occurred for the RRM1 fragment. Taken together, these results suggest that the linker between the RRM1 and RRM2 AUF1-p37^2RRMs^ proteins was cleaved by a certain protease, possibly a serine protease, and the free RRM1 fragment was specifically crystallized 1 or 2 days later.

### 3.4. Comparison with Other RRM-Containing Structures

**Figure 4:**
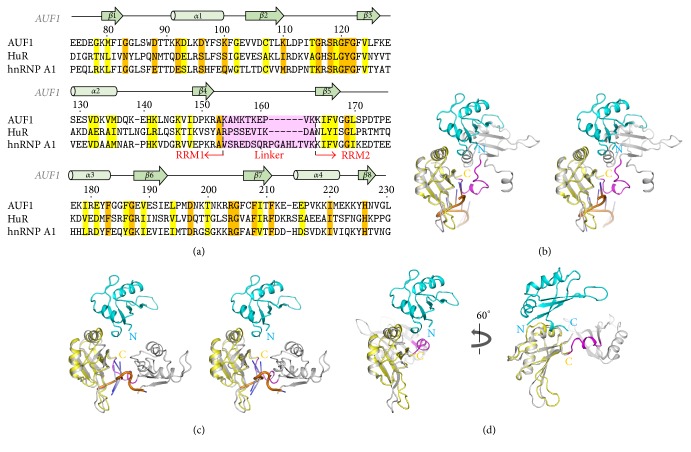
Comparison of the tandem RRM conformations. (a) Sequence alignment of tandem RRM1 and RRM2 regions of AUF1, HuR, and hnRNP A1. Conserved residues are presented in either orange or yellow backgrounds depending on their degree of conservation. The linker between RRM1 and RRM2 is highlighted in pink and also indicated under the sequences. The secondary-structure element of AUF1 is shown. (b) Stereo diagram of overlay of dimeric AUF1-RRM1 and hnRNP A1 in complex with telomeric DNA fragment (PDB entry 2UP1). The colors of the dimeric AUF1-RRM1 are the same as in Figure 2(b). The hnRNP A1 and DNA are colored gray and orange. The linker between RRMs is shown in magenta. (c) Stereo diagram of overlay of dimeric AUF1-RRM1 and HuR in complex with the AU-rich RNA fragment (PDB entry 4ED5). The colors of the dimeric AUF1-RRM1 are the same as in Figure 2(b). HuR and RNA are gray and orange, respectively. The linker between RRMs is shown in magenta. (d) Overlay of dimeric AUF1-RRM1 and HuR apo (PDB entry 4EGL). The 60° rotated view along the *y*-axis is shown on the right panel. The colors of the dimeric AUF1-RRM1 are the same as in Figure 2(b). HuR is colored gray. The linker between RRMs is shown in magenta.

To date, several crystal structures of hnRNP-related tandem RRMs have been reported, in either the apo or nucleic acid complex forms [33, 35, 36]. Although we only obtained the AUF1-RRM1 structure, it is still possible to compare the structure with other tandem RRM structures such as HuR and hnRNP A1, because two molecules of AUF1-RRM1 are found in the asymmetric unit. Furthermore, the C-terminus of RRM1-Mol. 1 is 25.8 Å from the N-terminus of RRM1-Mol. 2, which suggests their possible covalent linkage. To explore that possibility, we first compared the length of the linkers between RRM1 and RRM2 in AUF1, HuR, and hnRNP A1 (Figure 4(a)). The linkers in AUF1 and HuR were the same size, while that in hnRNP A1 was six residues longer. Next, to determine the conformational differences between RRM2 and Mol. 2, we superimposed the structure of RRM1 on those of AUF1, HuR, and hnRNP A1 (Figures 4(b)–4(d)). As expected, all the various conformations of RRM2 indicated the flexibility of the linker region. Thus, no extensive interactions were found between the linker and the RRMs. Moreover, the conformation of HuR-RRM2 was substantially changed by RNA binding [33] (Figures 4(c) and 4(d)), while there was no significant difference between the apoprotein and DNA-bound hnRNP A1. Taken together, these results suggest that even though Mol. 1 and Mol. 2 are not structurally connected, the arrangement of Mol. 1 and Mol. 2 found in the structure of AUF1-RRM1 might be one of the possible conformations of AUF1 oligomerization. Further investigation is necessary to determine whether conformational changes are induced by RNA binding or other intrinsic features of AUF1.

## 4. Conclusion

We obtained the AUF1-p37^RRMs^ protein by RNase treatment and determined the high resolution crystal structure of AUF1-RRM1. Although we unexpectedly observed the crystallographic dimer of RRM1, by limited proteolysis experiments we proved that the linker between RRM1 and RRM2 is sensitive to certain proteases, possibly serine proteases. Furthermore, by overlaying the dimeric RRM1 with other RRM-containing structures, we might provide insight into the possible conformation of the tandem RRM of AUF1.

## Figures and Tables

**Table 1:** Data collection and refinement statistics for AUF1-p37^RRM1^.

Statistics	Se-SAD
*Data collection*	
Wavelength (Å)	0.9796
Space group	*P*2_1_2_1_2_1_
Cell dimensions (Å)	
*a*, *b*, *c* (Å)	39.07, 39.41, 93.25
*α*, *β*, *γ* (°)	90, 90, 90
Resolution (Å)	50.00–1.70 (1.76–1.70)^a^
Total reflections	524815
Unique reflections	16559 (1565)
*R*_merge_^b^ (%)	9.3 (32.9)
*I*/*σ* (*I*)	42.2 (9.3)
Completeness (%)	99.9 (100.0)
Redundancy	7.1 (7.1)
*Phasing*	
Overall figure of merit	0.493
*Structure refinement*	
Resolution (Å)	26.6–1.70
Number of reflections	30748
*R*_work_^c^/*R*_free_ (%)^d^	16.98/20.40
Number of atoms; proteins/water	1280/263
RMS deviation	
Bond lengths (Å)	0.006
Angles (°)	0.99
Average *B*-factor (Å^2^)	19.90
Ramachandran plot (%)	
Favored region	100
Outliers	0
*PDB accession code*	5IM0

^a^The numbers in parentheses are statistics from the highest resolution shell.

^b^ *R*_merge_ = ∑|*I*_obs_ − *I*_avg_|/*I*_obs_, where *I*_obs_ is the observed intensity of individual reflection and *I*_avg_ is the average over symmetry equivalents.

^c^ *R*_work_ = ∑||*F*_*o*_| − |*F*_*c*_||/∑|*F*_*o*_|, where |*F*_*o*_| and |*F*_*c*_| are the observed and calculated structure factor amplitudes, respectively.

^d^ *R*_free_ was calculated from 5% of the data.
